# Comparative Study of Postoperative Outcomes of Clavicle Midshaft Fracture Treated by Nailing vs. Plating

**DOI:** 10.7759/cureus.22862

**Published:** 2022-03-05

**Authors:** Siddharth Yadav, Mukesh O Phalak, Ishan Shevate, Rahul Salunkhe, Ashwinkumar Khandge, Ashwin Deshmukh, Shivam Patel, Gaurav L Patil

**Affiliations:** 1 Orthopaedics, Dr. D.Y. Patil Medical College, Hospital & Research Centre, Pune, IND; 2 Orthopaedics and Trauma, Dr. D.Y. Patil Medical College, Hospital & Research Centre, Pune, IND; 3 Orthopaedics, Topiwala National Medical College and B.Y.L. Nair Charitable Hospital, Mumbai, IND

**Keywords:** titanium elastic nails, locking plate, union time, constant-murley score, functional outcome, clavicle fracture

## Abstract

Background

A midshaft clavicle fracture is a prevalent form of injury of the upper extremity that affects one's quality of life. Several treatment modalities facilitate fixation of the displaced midshaft clavicle to decrease nonunion and malunion of the clavicle fracture. Still, numerous factors influence choosing an optimal surgical intervention. Thus, this study investigates the functional outcome of two standard fixation techniques, titanium elastic nails (TENs) and locking plates, as a prospective comparative study for surgical management of displaced midshaft clavicle fractures.

Methods

We performed closed/open reduction and internal fixation in 62 patients (40 male and 22 female) with TENs and locking plates, respectively, which were followed up at regular intervals following the surgery (at two, six, 12, 24, and 48 weeks). The surgical outcome was assessed both from functional and radiological standpoints. The influence of surgical fixation on functional outcome was evaluated based on the Constant-Murley score and the fracture recuperation based on union times.

Results

When compared to plate fixation, TENs had lesser union times. Still, there was no statistical difference in union time between the two groups. The functional assessment graded by Constant-Murley score had a similar distribution of scores between the two groups.With a follow-up of twelve months, the Constant-Murley scores between the groups were not statistically different. While the average score for plate fixation was slightly higher than that of TENs, the nonunion rate was found to be similar in both groups.

Conclusion

Surgical interventions using both TENs and plate fixation are suitable for managing clavicle midshaft fractures as they have a similar functional outcome. However, considering early recovery with minimal surgical complications, TENs can be a preferred treatment choice for managing displaced midshaft clavicle fractures.

## Introduction

The clavicle is highly susceptible to fracture from impacts at the shoulder joint. Thus, it accounts for 44% of shoulder injuries and 2.6-10% of all fracture types [1,2]. Moreover, almost half of the clavicle fractures are displaced, and patients younger than forty years and older than seventy years are the predominant demographic to experience clavicle fractures from traffic accidents, sudden falls, and shoulder injuries [1,3].

The clavicle is more like a strut that facilitates proper upper limb function at the shoulder. Furthermore, it biomechanically transmits forces from the upper limb to the trunk. The superficial location of the clavicle at the shoulder, combined with its innately thin midshaft, makes them highly susceptible to injury while transmitting forces during impactful falls with the arm at the side. Based on the anatomical site of the fracture, clavicle fractures are commonly categorized using the Allman classification into Type I (fracture at the middle third), Type II (fracture at the lateral third), and Type III (fracture at the medial third). During an impact at the shoulder joint, the constricted cross-section of the clavicular midshaft experiences higher compressive stress levels to cause an imminent fracture. Thus, most clavicular fractures (80-85%) occur at the midshaft [1].

Clavicle fracture causes pain and swelling at the fracture site. The inability to lift or use the arm can significantly affect someone from carrying out their activities of daily living (ADL). Conservative treatments of clavicle fracture include the use of shoulder arm pouch, triangular sling, clavicle brace, and figure-of-eight bandages. Conservative management is still preferred for minimally displaced fractures [4]. However, for complex fractures (highly displaced or comminuted), these conservative methods can be ineffective and can result in subsequent malunion/non-union, distortion, and cosmesis [5-6]. Moreover, elderly patients and patients with comminuted fractures with no bony contacts or displacement tend to pose a higher risk [7]. Nevertheless, advances in operating techniques, metallurgy, and medical imaging have made surgical intervention effective for managing complex fractures. Treatment using K-wire fixation, Austin Moore pins, Steinmann pins, titanium elastic nails (TENs), and fixation plates are widely adopted. Moreover, studies reported that surgical interventions improved functional outcomes with higher union rates when compared to conservative methods [8-9].

Standard surgical treatment for a complex midshaft clavicular fracture involves open reduction and internal fixation (ORIF) using plates or intramedullary fixation using TENs. TENs are widely adopted as an alternative to a plate or screw fixation [10-12]. TENs have also been attempted as closed reduction and internal fixation (CRIF). It has been demonstrated to be a safe, minimally invasive technique that achieves primary stability functional and cosmetic outcomes. Compared to plate osteosynthesis, TENs facilitate rapid healing with few complications. Moreover, the stress distribution of TENs is similar to that of an intact clavicle [13]. Nevertheless, fixation using reconstruction plates had improved stress shielding and was more stable to facilitate early recovery to carry out ADL [13]. Also, the superiority of plate fixation over TENs was due to its resistance to bending and torsional forces [14]. Plate fixation also reduces non-union and symptomatic malunion rates, making it an ideal choice for treating displaced midshaft clavicle fracture [15]. Although TENs (intramedullary fixation) and plate fixation seem to be viable treatments for managing complex midshaft clavicle fracture, fracture characteristics, patient comorbidities, and functional outcome expectations must be considered while choosing an appropriate treatment [16].

This study aims to identify a suitable treatment for midshaft clavicle fracture by comparing internal fixation with TENs and locking plates based on their union time and functional outcomes. Additionally, this study also aims to examine the indications and contraindications of two fixing techniques and their drawbacks.

## Materials and methods

A prospective comparative study was conducted to assess the influence of two fixation techniques (TENs and locking plates) on the functional outcomes while managing displaced midshaft clavicle fracture.

This study was conducted at the department of orthopedics for one year, between Jan 2020 to Jan 2022. Informed consent was obtained from all participants prior to surgical procedures, and the institution's ethics approval committee approved the study. The study was approved by Institutional Review Board, Institutional Ethics Sub-committee of Dr. D.Y. Patil Medical College, Hospital and Research Centre, Pune, in its meeting held on 04/09/2019 with approval number IESC/PGS/2019/94.

The following inclusion and exclusion criteria were considered while recruiting patients for the study. Patients between 16 and 60 years of age, patients presenting mid-shaft clavicle fractures that are displaced, patients with bone shortening or overriding of more than two centimeters, and with bilateral clavicle fracture were included in the study. Patients less than 16 and above 60 years of age, patients with previously diagnosed pathology in the shoulder or elbow, patients with any pre-existing fracture lasting for more than two weeks, patients presenting with fracture of the clavicle at lateral and medial ends, patients with scapular malposition and winging upon initial examination, patients with floating shoulder and compound fractures of grade III and unwilling patients were excluded from the study.

Patients considered for the study underwent surgical procedures following the institution's surgical protocols. Pre- and postoperative assessments were carried out to evaluate their functional outcome. Lastly, statistical analysis was performed to assess the influence of the two fixation techniques before and after surgery. 

Patients underwent initial clinical assessments that required first aid, including a broad arm sling, oral or parenteral analgesics, and antacids. A definitive treatment plan was formulated based on radiographic assessments. X-rays with both anteroposterior (AP) and lordotic views (for the entire length of the clavicle) were obtained for all patients. Additionally, CT scans and MRI were performed if deemed necessary.

Prognostic classification of patients was done as per Allman classification. Patients were randomly assigned into two interventional groups and underwent surgery upon anesthetic and surgical readiness. The first group (group I) received TENs (as CRIF or ORIF) based on suitability, and the second group (group II) received only plate fixation (as ORIF). After general anesthesia, patients received surgical procedures and were placed supine on the operating table.

**Figure 1:**
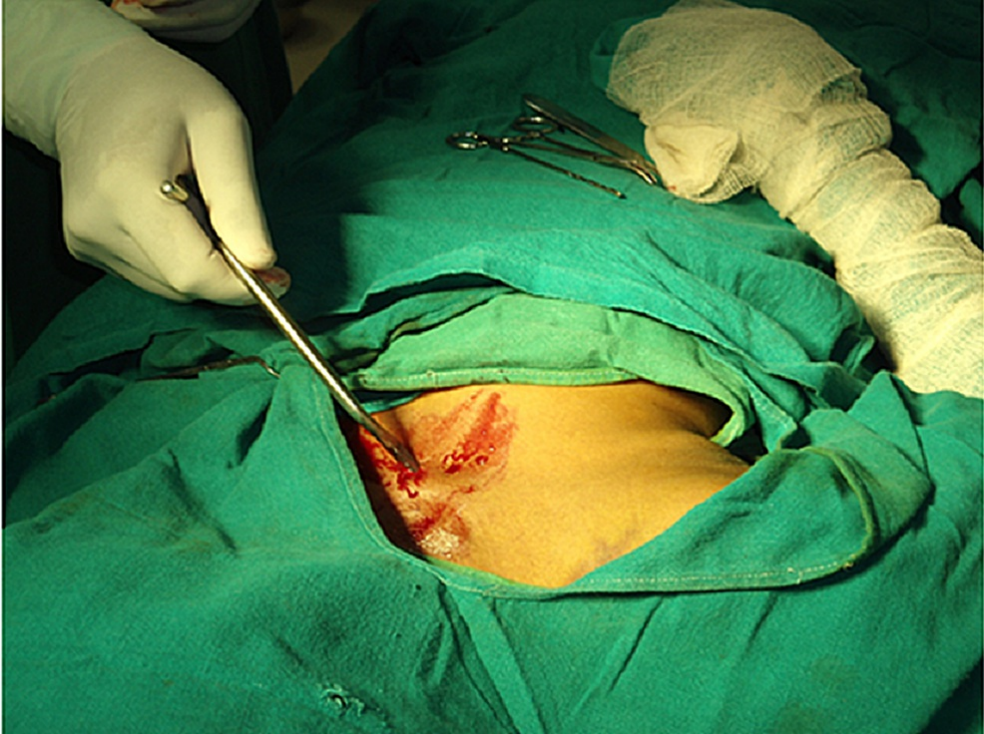
Surgical insertion of titanium elastic nail (TEN) into the fracture region, showing opening the anterior cortex using an awl

The sternoclavicular joint on the afflicted side was palpated and demarcated to make a tiny incision of one centimeter towards its side. Later, a sharply pointed awl was used to open the anterior cortex into which TENs were introduced, as shown in Figure 1. The diameter of TENs varied between two to three millimeters, which was chosen to fit the diameter of the medullary canal. Two-pointed reduction clamps were used to perform a closed fracture reduction under fluoroscopic guidance. Additionally, a mini-open incision was made over the fracture site to directly manipulate the fracture pieces for fixation if closed reduction failed. The TENs were steadily pushed until it was medial to the acromioclavicular joint, and the fracture was minimized/compressed at its terminus. Any penetration into the thin dorsal cortex was avoided by precisely maneuvering the nail tip under fluoroscopic guidance. The nail was cut near the entry point to allow enough length for simple removal if needed. Lastly, the skin layers and fascia were closed.

**Figure 2:**
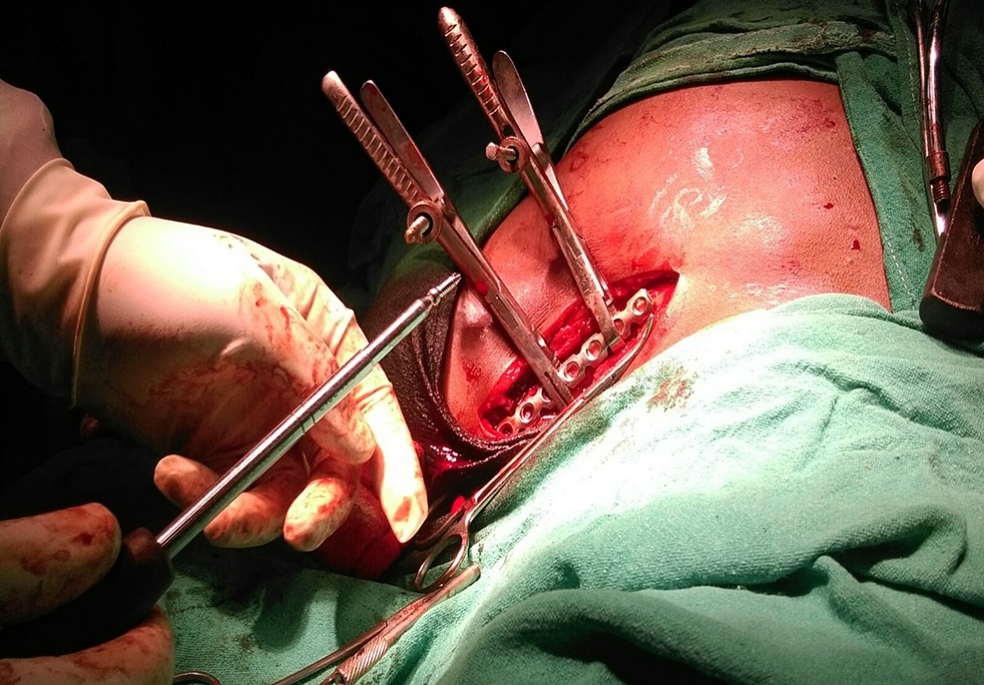
Intra-operative plate fixation after fracture reduction

When the patient was in the supine position, a sandbag was wedged between the medial border of the scapular and spine. A transverse incision was made over the fracture site. During this incision, the supraclavicular nerve was secured whenever possible. The fracture was reduced, and a plate of appropriate size was placed on the clavicle's anterosuperior surface, wherein screws secured the plates (Figure 2). If the fractures were identified to be oblique, interfragmentary compression was achieved using lag screws. Lastly, the skin layers and fascia were closed.

After surgery, patients were given a broad arm sling and were advised to begin early shoulder mobilization with pendular exercises on the second day. After seven days, active range of motion (ROM) exercises were initiated. After two weeks, overhead shoulder abduction was allowed.

**Figure 3:**
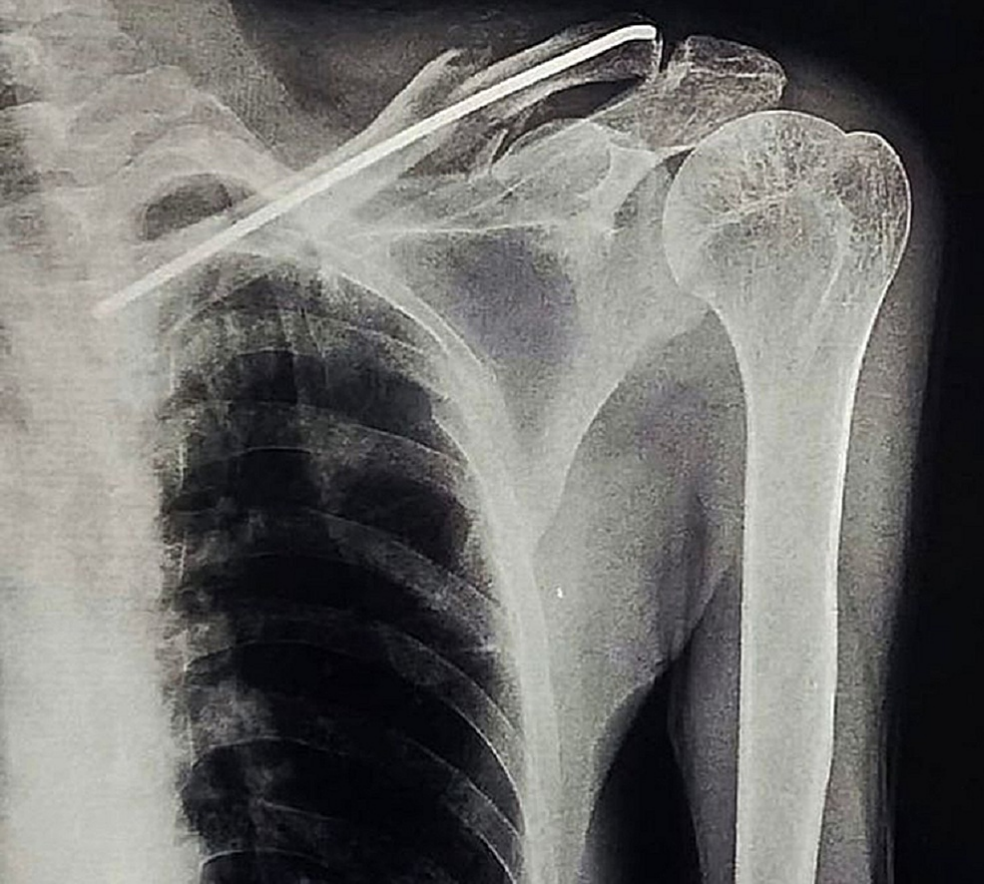
Postoperative X-ray of left clavicle fracture treated with intramedullary fixation using a titanium elastic nail (TEN)

**Figure 4:**
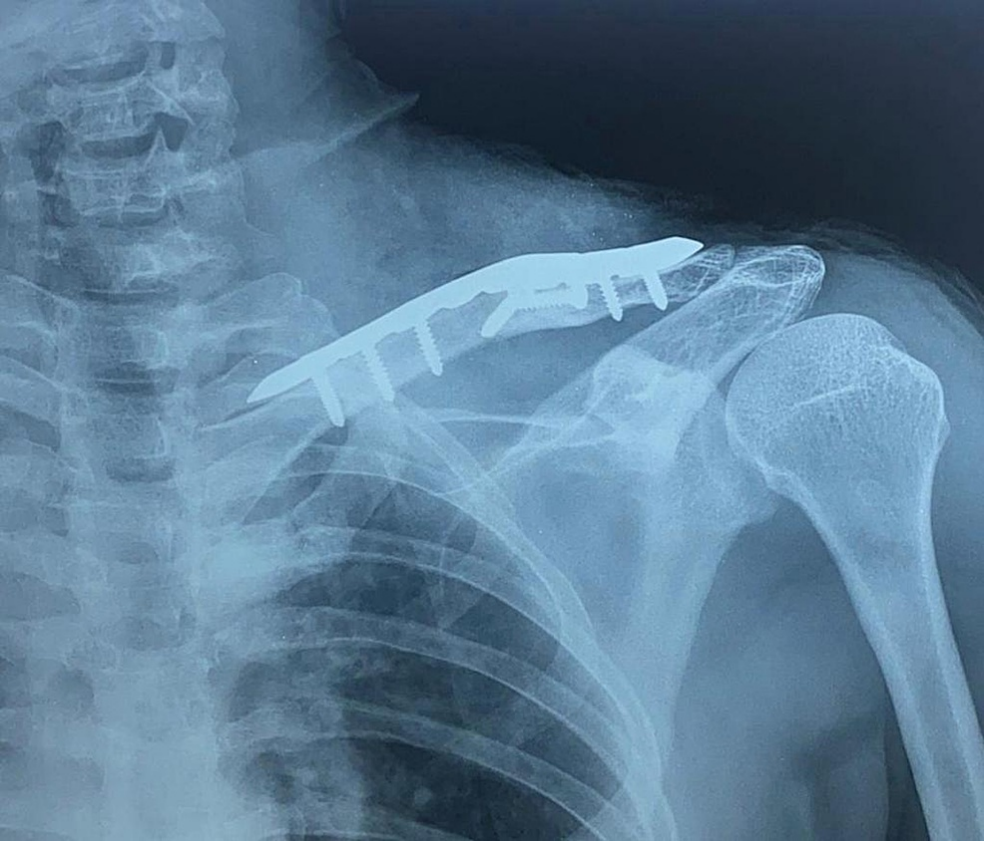
Postoperative X-ray of left clavicle fracture treated with plate fixation

Both functional and radiological assessments (Figures 3-4) were performed after two weeks, six weeks, three months, six months, twelve months, eighteen months, and twenty-four months to evaluate the overall surgical outcome. The modified Constant-Murley score [17], which included pain, strength, ADL, and ROM, quantified the functional outcome. Shoulder movements, including external rotation, abduction, internal rotation, and forward flexion measured by the ROM around the sternoclavicular and acromioclavicular joint. Radiographic assessments included the investigation of callus bridging and obliteration of fracture lines. The time taken for bridging of fracture (from radiographs) and pain mitigation were recorded. Additionally, the clavicle length between the sternal to the acromial end was measured between the operated and healthy shoulder.

Functional effects of TENs and plate fixation were inferred from statistical analysis performed using SPSS 17.0 software (SPSS, Inc., Chicago, IL, USA). Appropriate parametric (Student's t-test) and non-parametric tests (Fisher's exact test) were conducted to identify significant differences in functional outcomes between the two interventions.

## Results

In this prospective comparative study, the functional outcome and union time for TENs and plate fixation has been compared across sixty-two patients presenting with clavicle fracture. Among them, 40 patients (65%) were male, and the remaining were female. While most patients (48.39%) were between 21 and 30 years, the age range varied from 17 to 60 years. Furthermore, 42 (67.74%) patients had clavicle fractures from road accidents, 15 (24.19%) from falls, and five (8.06%) from assaults.

According to our inclusion criteria, only fractures in the middle third of the clavicle were considered in the study, while those at the medial and lateral ends were omitted. None of the patients had bilateral involvement, and the most prevalent form of fracture was the transverse type, which accounted for 66.13% of the total. Also, considering the nature of the fracture, 57 (91.94%) patients had a closed injury, and a very small proportion, five (8.06%) patients, had an open injury.

Patients were randomly assigned into two groups: those who received TENs (group I) and plate fixation (group II) to manage clavicle fracture. Each group had thirty-one patients, with both groups having a similar average age of thirty years. As seen in Table 1, 27 patients in group I underwent TENs as CRIF, and the remaining four patients had TENs as ORIF. All patients in Group II were treated with plate fixation as ORIF.

**Table 1:** Patient distribution for different surgical interventions treated using titanium elastic nailing (group I) and plate fixation (group II)

Procedure	Group I	Group II
Closed reduction and internal fixation (CRIF)	27 (87.09%)	-
Open reduction and internal fixation (ORIF)	4 (12.90 %)	31 (100%)

As seen in Table 2, when compared to group II that received plate fixation, the group I with TENs had fewer union times. Within 6-12 weeks postoperative, five patients in group I had fracture union. Moreover, 27 patients (87.09%) in group I experienced fracture union between 12-24 weeks, whereas only 16 (51.6%) patients had fracture union in group II. A fracture is considered to be non-union if there is no recuperation between six and nine months after the damage, which was the same between the groups.

**Table 2:** Comparing the status of fracture union and union time between titanium elastic nailing (group I) and plate fixation (group II)

Status of union	Time of union (in weeks)	Group I	Group II
United	<6	1 (3.22%)	-
	6-12	5 (16.12%)	0 (6.45%)
	12-18	10 (32.26%)	6 (12.90%)
	18-24	12 (38.71%)	10 (32.25%)
	24-36	1 (25.80%)	13 (41.94%)
Non-united	Up to 48	2 (6.45%)	2 (6.45%)

The functional assessment graded by the Constant-Murley score shows the difference between normal and abnormal sides (Table 3). The distribution of scores was very similar between the two groups, where 29 (93.55%) patients had an excellent functional outcome. While the remaining two patients from group II had a good functional outcome, for group I, one had a poor, and the other had a good functional outcome.

**Table 3:** Comparing the functional outcome (graded by Constant-Murley score) between titanium elastic nailing (group I) and plate fixation (group II)

Poor		Fair		Good		Excellent	
Group I	Group II	Group I	Group II	Group I	Group II	Group I	Group II
1 (3.23%)	-	-	-	1 (3.23%)	2 (6.45%)	29 (93.55%)	29 (93.55%)

During the follow-up period of twelve months, the Constant-Murley scores were not statistically different between the two groups (Table 4). Still, the average score for group II (95.45+4.28) was slightly higher than for group I (94.19+8.88). Moreover, the deviation of Constant-Murley scores from its average for group I (SD=8.88) was twice that of group II (SD= 4.28). 

**Table 4:** Postoperative assessment after twelve months comparing the Constant-Murley score between titanium elastic nailing (group I) and plate fixation (group II)

Assessment period	Group I	Group II	p-value (not significant)
12 months	94.19+8.88	95.45+4.28	0.25

As seen in Table 5, plate fixation had greater intraoperative and postoperative problems than TENs, including more blood loss, more operative time, superficial and deep infections, implant protuberance, unsightly scars, and difficult implant removal.

**Table 5:** Surgical complications across patients who received titanium elastic nailing (group I) and plate fixation (group II)

Complications	Group I	Group II
Superficial infection	5 (16.12 %)	3 (9.67 %)
Deep infection	-	2 (6.45 %)
Neurovascular injury	-	-
Non-union	2 (6.45 %)	2 (6.45 %)
Ugly scar	-	3 (9.67 %)
Implant protuberance	-	2 (6.45 %)
Pin migration	2 (6.45 %)	-
Implant failure	-	-
No complication	22 (67.74%)	19 (61.29%)

## Discussion

Sixty-two patients with clavicle fractures were randomly separated into two interventional groups - those who received TENs and plate fixation. With two-thirds of the patients being male, this uneven gender distribution in our study was supported by existing literature where most patients presented with clavicle fracture were male [1,12,18-20]. Moreover, the observation from our study that younger individuals aged between 21 and 40 years were susceptible to clavicle fracture was consistent with other studies [1,3,12,18-20]. Young patients are more prone to fractures of the middle third of the clavicle due to their involvement in heavy jobs and outdoor activities, making them more susceptible to these injuries. In our study, the most common mode of injury was a motor vehicle accident, followed by a fall from a height and assault. This also explains why males outnumber females as males are more vulnerable to these injuries than females, as males are more involved in outdoor activities.

The treatment of clavicle fracture was typically conservative until the twentieth century. However, fracture reduction was minimal with conservative methods and often resulted in malunion/non-union. On the contrary, closed/open reduction of these fractures and internal fixation tend to overcome the drawbacks of conservative methods. Thus, the study aimed to compare two widely adopted fixation techniques.

Upon comparing the healing time, the early formation of callus facilitated swift healing in group I. Still, non-union at the fracture site was observed across the same number of patients in both the groups. A similar observation of faster healing and union in patients treated by the intramedullary nail as compared to plate fixation was reported by other studies [10,21-23]. Plate fixations tend to provide rigid stabilization than TENs and had better fracture management for complex clavicle fractures by applying the bridge plating technique [13]. However, such a procedure requires large incisions and can injure soft tissues to cause several postoperative complications. Thus, intramedullary fixation (TENs) stands out as a minimally invasive alternative to plate fixation. Furthermore, TENs offer stable fixation allowing axial compression and enhancing healing by preserving the integrity of soft tissues, membranes of the bone surface, and vascular construct at the fracture site.

This study observed a higher occurrence of superficial infection among group I (TENs) patients, where a well-defined protocol of wound assessment and management along with antibiotics was followed. However, none of the patients had deep infections at the operating site when treated by TENs. Still, other complications, including ugly scar, implant protuberance, pin migration, and non-union, were encountered. Nevertheless, TENs allowed early relief from shoulder pain and improved cosmesis. Additionally, with the progression of callus formation, any underlying infection may reduce. In our study, the plating group had more complications than the TENs group, as per available literature [13,24-25].

In our study, the overall functional outcome evaluated using the Constat-Murley scores were similar between the two groups. Moreover, similar to other reported studies [23,26], regardless of early recovery amongst patients receiving TENs, there was no significant difference in the mean scores after one-year postoperatively. Compared to ORIF by plate fixation, patients treated with CRIF by TENs had shorter operative time, less time at the hospital (postoperative recovery), and a faster fracture union. These observations were also reported in similar studies [10,21-22]

## Conclusions

This study compared surgical intervention via two fixation techniques to manage displaced mid-shaft clavicle fracture across sixty-two patients. In terms of the functional outcome, the Constant-Murley scores had no significant difference between TENs and plate fixation. Similarly, there was no statistical difference in union time. Moreover, non-union was equal in both TENs and plate fixation groups. Faster healing was observed with TENs when compared to plate fixation as the callus were formed early. Still, anatomical alignment, the rigidity of fixation, and compression at the fracture site were better in plate fixation. Nevertheless, plate fixation as an overall surgical intervention tends to have several intra-and postoperative complications, including blood loss, infections, implant protuberance, scarring, and longer operative time when compared to TENs. Regardless of any significant differences between TENs and plate fixation, considering the secondary outcomes of early recovery and minimal complications, TENs can be a preferred treatment choice for managing displaced midshaft clavicle fractures (Type I of Allman classification).

## References

[1] Postacchini F, Gumina S, De Santis P, Albo F (2002). Epidemiology of clavicle fractures. J Shoulder Elbow Surg.

[2] O'Neill BJ, Hirpara KM, O'Briain D, McGarr C, Kaar TK (2011). Clavicle fractures: a comparison of five classification systems and their relationship to treatment outcomes. Int Orthop.

[3] Kim W, McKee MD (2008). Management of acute clavicle fractures. Orthop Clin North Am.

[4] De Giorgi S, Notarnicola A, Tafuri S, Solarino G, Moretti L, Moretti B (2011). Conservative treatment of fractures of the clavicle. BMC Res Notes.

[5] Neer CS (1963). Fracture of the distal clavicle with detachment of the coracoclavicular ligaments in adults. J Trauma.

[6] Neer CS (1960). Nonunion of the clavicle. J Am Med Assoc.

[7] Nowak J, Holgersson M, Larsson S (2004). Can we predict long-term sequelae after fractures of the clavicle based on initial findings? A prospective study with nine to ten years of follow-up. J Shoulder Elb Surg.

[8] Canadian Orthopaedic Trauma Society (2007). Nonoperative treatment compared with plate fixation of displaced midshaft clavicular fractures. A multicenter, randomized clinical trial. J Bone Joint Surg Am.

[9] Buckley R, Leighton R, Trask K (2011). The Canadian Orthopaedic Trauma Society. J Bone Joint Surg Br.

[10] Mueller M, Rangger C, Striepens N, Burger C (2008). Minimally invasive intramedullary nailing of midshaft clavicular fractures using titanium elastic nails. J Trauma.

[11] Narsaria N, Singh AK, Arun GR, Seth RR (2014). Surgical fixation of displaced midshaft clavicle fractures: elastic intramedullary nailing versus precontoured plating. J Orthop Traumatol.

[12] Mishra PK, Gupta A, Gaur SC (2014). Midshaft clavicular fracture and titanium elastic intra-medullary nail. J Clin Diagn Res.

[13] Zeng L, Wei H, Liu Y (2015). Titanium elastic nail (TEN) versus reconstruction plate repair of midshaft clavicular fractures: a finite element study. PLoS One.

[14] Golish SR, Oliviero JA, Francke EI, Miller MD (2008). A biomechanical study of plate versus intramedullary devices for midshaft clavicle fixation. J Orthop Surg Res.

[15] McKee MD (2010). Clavicle fractures in 2010: sling/swathe or open reduction and internal fixation?. Orthop Clin North Am.

[16] Poigenfürst J, Rappold G, Fischer W (1992). Plating of fresh clavicular fractures: results of 122 operations. Injury.

[17] Constant CR, Murley AHG (1987). A clinical method of functional assessment of the shoulder. Clin Orthop Relat Res.

[18] Oliveira AS Junior, Roberto BB, Lenza M (2017). Preferences of orthopedic surgeons for treating midshaft clavicle fracture in adults. Einstein.

[19] Madhukar K, Sateesh GS (2015). A comparative study of clavicle fractures by conservative and operative management. Indian J Public Health Res Dev.

[20] Pranav VM, Chishti SN, Singh SN, Maske R, Soring D, Parija D (2016). A prospective study of operative management of simple midshaft clavicular fracture with titanium elastic nail (TEN). Int J Orthop Sci.

[21] Wu CC, Shih CH, Chen WJ, Tai CL (1998). Treatment of clavicular aseptic nonunion: comparison of plating and intramedullary nailing techniques. J Trauma.

[22] Hartmann F, Hessmann MH, Gercek E, Rommens PM (2008). Elastic intramedullary nailing of midclavicular fractures. Acta Chir Belg.

[23] Liu HH, Chang CH, Chia WT, Chen CH, Tarng YW, Wong CY (2010). Comparison of plates versus intramedullary nails for fixation of displaced midshaft clavicular fractures. J Trauma.

[24] Kadakia AP, Rambani R, Qamar F, McCoy S, Koch L, Venkateswaran B (2012). Titanium elastic stable intramedullary nailing of displaced midshaft clavicle fractures: a review of 38 cases. Int J Shoulder Surg.

[25] Gao Y, Chen W, Liu YJ, Li X, Wang HL, Chen ZY (2016). Plating versus intramedullary fixation for mid-shaft clavicle fractures: a systemic review and meta-analysis. Peer J.

[26] Saha P, Datta P, Ayan S, Garg AK, Bandyopadhyay U, Kundu S (2014). Plate versus titanium elastic nail in treatment of displaced midshaft clavicle fractures: a comparative study. Indian J Orthop.

